# Further Evidence of Emotional Allodynia in Unmedicated Young Adults with Major Depressive Disorder

**DOI:** 10.1371/journal.pone.0080507

**Published:** 2013-11-28

**Authors:** Alexander Ushinsky, Lindsay E. Reinhardt, Alan N. Simmons, Irina A. Strigo

**Affiliations:** 1 University of California San Diego, La Jolla, California, United States of America; 2 Center of Excellence for Stress and Mental Health at the Veterans Affairs San Diego Healthcare System, San Diego, California, United States of America; 3 BioCircuits Institute (BCI), La Jolla, California, United States of America; New York University, United States of America

## Abstract

**Background:**

Recent evidence suggests that sensitivity to the emotional sequela of experimental thermal pain(measured by emotional unpleasantness) is heightened in individuals with major depressive disorder(MDD), a phenomenon we termed “emotional allodynia”. The aim of this study was to examine whether acute happy and sad mood induction alters emotional allodynia in MDD. We hypothesized that emotional allodynia will be a robust characteristic of individuals with MDD compared to healthy controls. Thus, it would remain following acute mood induction, independent of valence.

**Methods:**

Twenty-one subjects with current MDD and 21 well-matched healthy subjects(HC) received graded brief temperature stimuli following happy and sad mood inductions procedures(MIP). All subjects rated the intensity and affect(pleasantness/unpleasantness) of each stimulus. Sensory(pain intensity) and affective(pain unpleasantness) thresholds were determined by methods of constant stimuli.

**Results:**

The MIPs reliably induced happy and sad mood and the resulting induced mood and subjective arousal were not different between the groups at the time of temperature stimulation. Compared to HC, MDD individuals demonstrated emotional allodynia. We found significantly decreased affective pain thresholds whereby significantly lower temperatures became unpleasant in the MDD compared to the HC group. This was not observed for the sensory pain thresholds. Within the MDD, the affective pain thresholds were significantly lower than the corresponding pain intensity thresholds, whereby non-painful temperatures were already unpleasant for the MDD irrespective of the induced mood. This was not observed for the HC groups where the affective and pain intensity thresholds were comparable.

**Conclusions:**

These findings suggest that emotional allodynia may be a chronic characteristic of current MDD. Future studies should determine if emotional allodynia persists after psychological or pharmacological interventions. Finally, longitudinal work should examine whether emotional allodynia is a result of or vulnerability for depression and the role it plays in the increased susceptibility for pain complaints in this disorder.

## Introduction

Pain is a subjective experience which can be assessed along two dimensions, i.e., sensory pain intensity, which describes the discrimination of the stimulus intensity, and affective pain unpleasantness, which describes the emotional impact of the stimulus [1], [2]. In healthy individuals during brief experimental stimuli these dimensions are highly correlated [3]–[5]. While depression seems to alter the experience of pain, the direction and quality of this change is unclear. Some studies have found that individuals with depressed mood show increased sensory pain intensity thresholds (i.e., decreased experimental pain sensitivity) compared to healthy controls [6]–[11]. Conversely, others find that depressed individuals compared to non-depressed individuals show decreased pain thresholds and therefore increased sensitivity to experimental pain, especially when the emotional dimension of experimental pain is evaluated [12]–[18]. Consistent with the latter, our prior work showed that young adults with Major Depressive Disorder (MDD) reported increased affective response to brief temperature stimulation compared to matched, never depressed adults. We have termed this notion as “emotional allodynia” in depression, i.e., a qualitatively altered negative emotional response to normally non-aversive stimuli or pain affect without pain sensation [19]. This was motivated by two observations: 1) the temperatures that were not unpleasant to the controls were highly unpleasant to the depressed participants (i.e., the affective thresholds were significantly lower in depressed compared to controls); and 2) the temperatures that were not painful to the depressed subjects were already highly unpleasant (i.e., the affective thresholds were significantly lower than the corresponding intensity thresholds in depressed subjects). Thus depressed individuals were emotionally allodynic on both, the between- and within-group comparisons. This has recently been further supported using the thermal illusion in mildly depressed adults [18].

There is not a clear understanding whether abnormal pain sensitivity in MDD is a chronic or acute mood-dependent phenomenon. A prior study of acute sad and neutral mood induction in healthy controls showed deceased heat pain unpleasantness thresholds during sad mood [20]. Conversely, increased pain unpleasantness during sad mood compared to happy mood induction has been found in healthy controls [21]. However, these studies did not investigate how subjects with major depression are differentially responsive to mood induction during pain, in contrast to healthy volunteers. A study of depressed subjects and healthy controls found that sensory heat pain thresholds were similar in both groups following acute induction of sad mood [22]. This study suggests that acute mood overrides chronic mood changes during perception of pain. However, this study did not look to make a distinction between pain intensity and affective unpleasantness. Furthermore, since neutral mood is still perceived as more negative during a depressed mood [22], the neutral mood induction may not create a sufficient differential mood to evaluate the effect of valance on the interaction between chronic and acute mood effects on pain perception.

Thus, the aim of this study was to further investigate emotional allodynia in MDD by determining whether it is a robust phenomenon of MDD, or whether it is explained by acute mood states. Induction of acute sad moods increases the perception of pain [18], [23]–[25]; thus, the abnormal affective experience of pain in MDD may very well be influenced by the lower baseline moods in this disorder. The current study builds upon our prior findings of emotional allodynia in young adults with MDD [19] by examining the effect of acute mood induction in a new, larger cohort of MDD individuals, and the degree to which sensory (i.e., pain intensity) and affective (i.e., unpleasantness) thresholds are altered by acute induction of happy and sad mood. We hypothesized that emotional allodynia is a chronic phenomenon and that the acute mood induction will fail to overcome chronic mood states in depression and have no effect on the perception of pain, i.e., depressed individuals will still demonstrate the emotional allodynia.

## Materials and Methods

### Ethics Statement

All subjects provided written informed consent before participation in this study. Both the consent and study procedures were approved by the University of California San Diego Human Research Protection Program and Veterans Affairs San Diego Healthcare System Research & Development Committee.

### Subjects

Twenty-one unmedicated subjects with current Major Depressive Disorder (MDD) (11F, 10M) and twenty-one healthy control (HC) (11F, 10M) subjects gave written informed consent to participated in all aspects of this study (**Table 1**). Additionally, five MDD (1M) and two control (2M) subjects were consented, but were unable to complete study procedures due to equipment failure (4MDD, 1HC), inability to complete study procedures (1MDD) or scheduling conflict (1HC), and thus are not included in the study sample. The groups did not differ significantly in proportion of non-completers (Yates-corrected χ^2^ = 0.413, p = 0.520). Healthy control subjects did not significantly differ from MDD participants on age (t(40) = 1.17, p = 0.25), race (χ^2^ = 0.17, p = 0.98), education (t(40) = 0.30, p = 0.76), and gender (χ^2^ = 0, p = 1.0) (**Table 1**).

**Table 1:** Demographic, Clinical and Psychological Variables.

	MDD (11F; 10M)		HC (11F; 10M)		Stats	
	Mean	SD	Mean	SD	t/χ2	P
**Demographic Variables**						
Age (yrs)	29.5	8.9	26.3	8.4	1.17	0.25
Education (yrs)	15.1	2.1	14.9	2.0	0.30	0.76
**Marital Status**					2.42	0.30
Married/living w/ partner	N = 4		N = 3			
Never married	N = 15		N = 18			
Separated/divorced	N = 2		N = 0			
**Race/Ethnicity**					0.17	0.98
African American	N = 2		N = 2			
Caucasian	N = 7		N = 7			
Hispanic	N = 7		N = 6			
Asian/Other	N = 5		N = 6			
**Clinical Variables**						
**Comorbid Diagnosis**						
GAD	N = 1		NA			
Panic Disorder	N = 3		NA			
PTSD	N = 1		NA			
**MDD History**						
MDEs including current	3.9	4.6	NA			
Duration current MDE (mo)	8.0	12.8	NA			
Age of onset MDD (yr)	22.9	8.3	NA			
BDI-2	27.5	9.0	1.2	2.0	13.08	<0.01

MDD/(E) – Major Depressive Disorder/(Episode); HC – Healthy Control; BDI – Beck Depression Inventory 2;

In order to establish current and past psychiatric diagnoses, each subject underwent a Structured Clinical Interview for DSM-IV (SCID) [26], which was administered by trained interviewers. The Beck Depression Inventory, second edition (BDI-2) [27] was administered to quantify current depressive symptom severity. Subjects were excluded if they had taken psychiatric medications within the 30 days of study participation. MDD subjects reported an average of four lifetime Major Depressive Episodes (**Table 1**). Healthy control subjects had no history of MDD or other current or lifetime Axis-I psychiatric disorders. Among the MDD subjects, two also met criteria for current Panic Disorder (PD), one met criteria for current PD with agoraphobia and Post Traumatic Stress Disorder (PTSD), and one met criteria for current Generalized Anxiety Disorder (GAD).

Subjects were excluded from the study if they: a) fulfilled DSM-IV criteria for a history of alcohol/substance dependence of over 2 years or abuse within the past 30 days of study participation; b) used psychotropic medication within the last 30 days; c) fulfilled DSM-IV criteria for current or prior bipolar or psychotic disorder; d) had ever experienced a significant head injury; e) had clinically significant comorbid medical conditions such as cardiovascular and/or neurological abnormality, or any active serious medical problems requiring interventions or treatment; f) had a chronic pain disorder; or g) were experiencing active suicidal ideation.

### Experimental Paradigm

**Figure 1:**
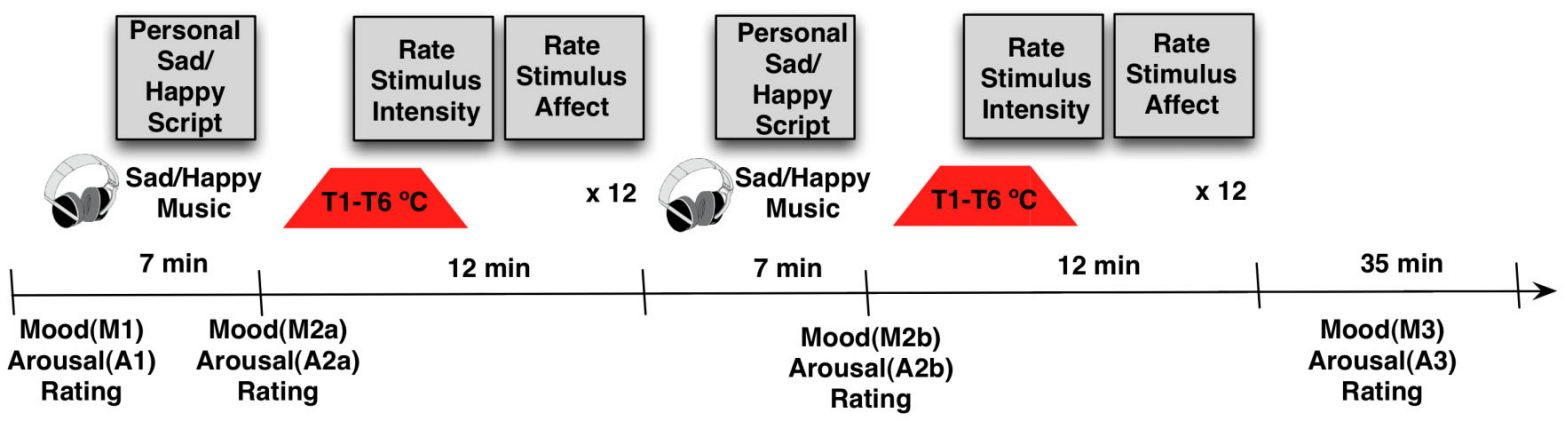
Experimental Design. Upon arrival subjects rated their mood and arousal (Rating 1). Subjects were then seated quietly in front of the laptop computer while listening to 7 min of previously chosen and individualized for each participant sad/happy music (see Appendix) and reading and re-experiencing the autobiographical event displayed on the computer screen. Mood and arousal ratings were taken immediately after (Rating 2a). This was followed by a series of brief temperature stimuli of six different intensities (T1–T6: 36, 38, 40, 42, 44, 46°C) delivered every 60 sec in random and counterbalanced order to subjects’ left forearm. After 12 temperature stimulations, mood induction was repeated with the new sad/happy music and autobiographical script, after which subjects again rated their mood and arousal (Rating 2b). A total of 24 temperature stimuli were delivered for a total duration of ∼40 min. After mood neutralization for ∼35 min (see Methods) subjects are asked to rate their mood and arousal (Rating 3). The procedure is repeated with the opposite valence.

This study used a validated Mood Induction Procedure (MIP) combined with brief temperature stimulation. The MIP, which combined 7 minutes of autobiographical recall and music (*see File S1*), has been previously shown to effectively induce both acute sad [28] and happy [29] moods. Brief temperature stimuli were administered using a procedure similar to the methods used previously by our lab in young adults with current MDD and HC subjects [19] (see below). A schematic of the computerized experimental paradigm is shown in **Figure 1**. All subjects underwent both happy and sad stimulation blocks on the same day, the order of which was pseudorandomized and counterbalanced between and within the groups. Each block consisted of MIP (sad, or happy), which was followed by the administration of brief temperature stimuli. Halfway through each block (sad or happy) a second MIP (a “booster”) of the same mood was given to ensure continuation of the mood. After each mood induction, subjects rated their mood and arousal [30] to evaluate the success of the MIP using the following scales: a) –10/“Extremely Sad” to +10/“Extremely Happy” with 0/“Neutral” as the midpoint; and b) –10/“Extremely Calm” to +10/“Extremely Aroused” with 0/“Neutral” as the midpoint, respectively. In addition, subjects used paper VAS before and after each experimental block in order to measure their baseline mood and to ensure that the mood returned to baseline level before continuing to a second block of opposite valence(**Figure 1**). The following paper VAS were used: a) –10/“Extremely Sad” to +10/“Extremely Happy” with 0/“Neutral” as the midpoint; and b) –10/“Extremely Calm” to +10/“Extremely Aroused” with 0/“Neutral” as the midpoint. During the procedure, each subject was alone in the testing room, was wearing headphones and seated comfortably in front of the laptop computer with a thermode attached to the left forearm. Between the sad and happy experimental blocks subjects took a break and were shown a neutral distraction video (*see File S1*) to minimize carryover effects between the moods [31], [32] **.**

**Temperature Stimulation and Thresholding.** Temperature sensitivity was assessed after each MIP using a 9 cm^2^ thermode (Medoc TSA-II NeuroSensory Analyzer, Ramat-Yishai, Israel). The thermode was applied to each subject’s left volar forearm after pretesting for tolerability. Temperature stimulation sequences consisted of randomly delivered short heat pulses (i.e., 5 seconds excluding rise/fall time) at six different temperatures (i.e., 36°C, 38°C, 40°C, 42°C, 44°C, and 46°C). Each temperature was administered four times, totaling 24 temperatures delivered in each mood type. Inter-stimulus interval time was 60 seconds to minimize habituation. Following each temperature stimulus, subjects were asked if they felt the stimulus, and if so to rate: a) its intensity (by indicating the maximum sensation of heat/pain on a scale from –10/“Neutral” to +10/“Extremely Painful” with 0/“Pain threshold” as the midpoint); and b) its affect (on a scale from –10/“Extremely Pleasant” to +10/“Extremely Unpleasant” with 0/“Unpleasantness Threshold” as the midpoint) using computerized VAS. Examination of subjects’ ratings showed that subjects did not perceive the majority of low temperature stimuli (i.e., 36°C and 38°C), so data are reported for the four temperature intensities that were consistently perceived. Stimulus-response curves for the intensity and unpleasantness ratings from these four temperatures were constructed for each individual. The intercepts from the linear regression lines were used as estimates of individual intensity and affective pain thresholds.

### Statistical Analyses

The mood induction order was entered as a covariate. First, in order to evaluate the effects of the mood induction procedure on subjects’ mood we used a two-way repeated measures ANOVA with group (MDD, HC) as a between-subject factor, and mood rating before induction (M1), immediately after induction (M2), and at the end of sad/happy block (M3) as a within-subjects repeated factor. The M2 factor was obtained by averaging mood ratings after the two mood inductions within each mood type (i.e., M2a and M2b, **see** **Figure 1**). Sad and happy mood inductions were entered into separate ANOVA models. Second, a similar analysis was conducted to evaluate the effects of mood induction on subjects’ arousal ratings with group as a between-subject factor, rating A1, A2 and A3 as a within-subjects repeated factor. Again, A2 was obtained by averaging arousal ratings after the two mood inductions within each mood type (i.e., A2a and A2b, see **Figure 1**). Significant between-subjects main effects were investigated by post-hoc two-way univariate ANOVAs. Third, in order to compare sensory and affective thresholds between the groups, we used univariate two-way ANOVA model with group as a between-subject factor, and mood (happy, sad), as a within-subjects repeated factor. Sensory and affective thresholds were entered into separate ANOVA models. Finally, a repeated measures ANOVA was used to compare sensory and affective pain thresholds within each group. All statistical analyses were performed with PASWstatistics 18.0 package (Chicago, IL).

## Results

### Mood Ratings

**Figure 2:**
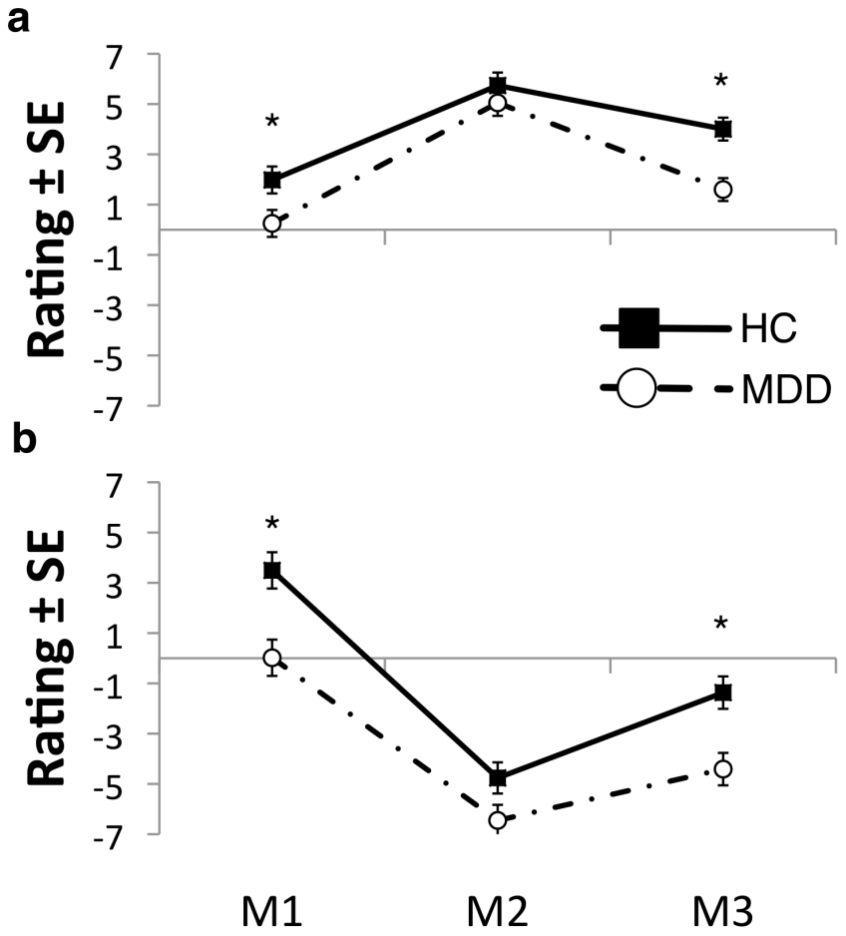
Mood Ratings. Subjects rated their mood prior to mood induction (M1), immediately after mood induction (M2) when temperature stimulations were applied and at the end of sad/happy experimental blocks (M3) (see **Figure 1** and text for more details). Significant within-subjects effects of mood induction on subjects’ rating of mood in both manipulations (happy: F(2,38) = 6.113, p<0.01; sad: F(2,38) = 12.306, p<0.001) with mood ratings being significantly lower during sad than happy MIP. (**A**) **Happy MIP**: MDD subjects showed significantly lower M1 (F(1,39) = 5.199, p<0.05) and M3 ratings (F(1,39) = 13.970, p<0.01) but not M2 rating (F(1,39) = 0.894, p>0.05); (**B**) **Sad MIP:** MDD subjects showed significantly lower M1 (F(1,39) = 11.584, p<0.01) and M3 (F(1,39) = 11.133, p<0.01) rating but not M2 rating (F(1,39) = 3.720, p>0.05).

Using subjects’ ratings of mood over time as a repeated measure in an ANOVA model showed significant within-subjects effects of mood induction on subjects’ ratings of mood in both manipulations (happy: F(2,38) = 6.113, p<0.01; sad: F(2,38) = 12.306, p<0.01) with mood ratings being significantly lower during sad than happy MIP. These results suggest that the MIPs were effective in inducing the desired mood in both groups. Significant group effect on subjects’ mood rating was also observed (happy: F(1,39) = 10.544, p<0.01; sad: F(1,39) = 13.275, p<0.01) with mood ratings overall being lower in the MDD than the HC group. Post-hoc investigation of these group effects showed that during both happy and sad MIP blocks, MDD subjects showed lower mood ratings before and after (F’s(1,39)>3.0; p<0.05), but not during (F’s(1,39)<3.0, p>0.05) mood inductions (**Figure 2a,b**). These results suggest that when brief temperatures were applied, the groups were not significantly different in their state mood rating.

### Arousal Ratings

**Figure 3:**
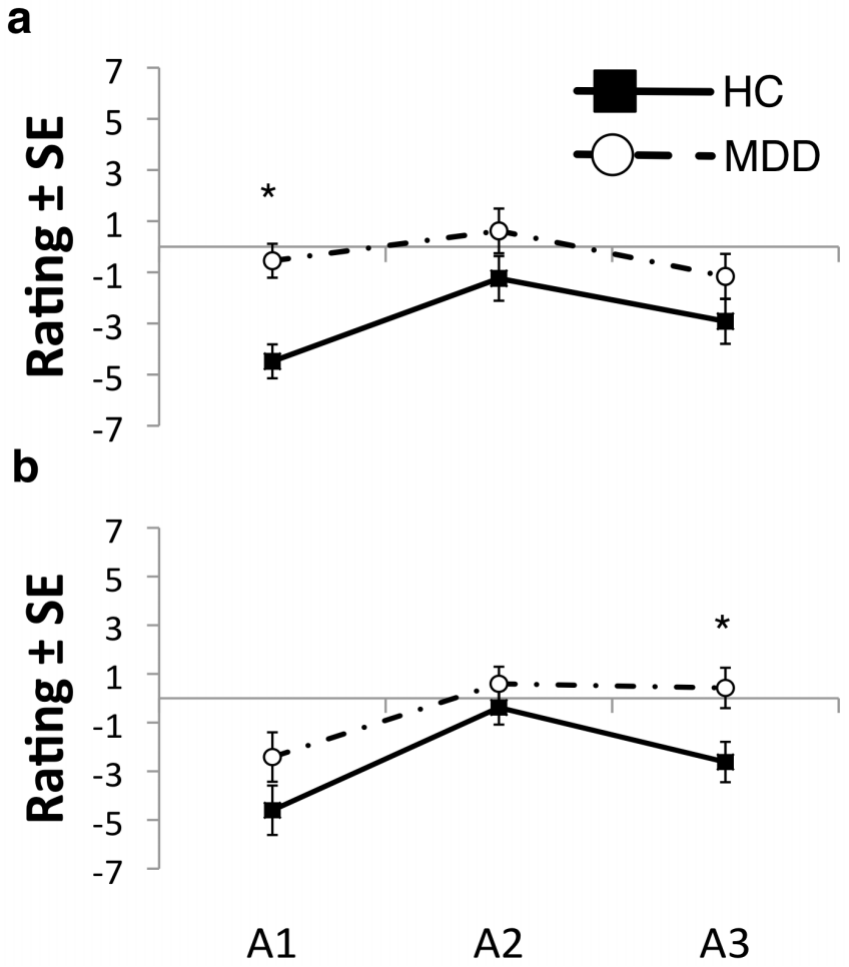
Arousal Ratings. Subjects rated their arousal prior to the mood induction (A1), immediately after mood induction (A2) when temperature stimulations were applied and at the end of sad/happy experimental blocks (A3) (see **Figure 1** and text for more details). ) **(a)** **Happy MIP**: MDD compared to controls had higher A1 rating (F(1,39) = 9.242, p<0.01) but not A2 (F(1,39) = 2.263, p>0.05) or A3 (F(1,39) = 1.981, p>0.05) rating. **(b)** **Sad MIP:** MDD compared to control group did not differ in their A1 (F(1,39) = 2.321, p>0.05) or A2 (F(1,39) = 0.972, p>0.05) rating and had higher A3 rating (F(1,39) = 6.735, p<0.05).

Using subjects’ ratings of arousal over time as a repeated measure in an ANOVA showed no significant within-subjects effects of mood induction on subjects’ ratings of arousal in either manipulation (happy: F(1,38) = 0.773, p = 0.469; sad: F(1,38) = 0.864; p = 0.430). These results suggest that arousal stayed relatively constant during the experimental procedure in both groups. Significant between-subjects effect of group on subjects’ arousal ratings was observed (happy: F(1,39) = 7.512, p<0.01; sad: F(1,39) = 4.914, p<0.05) with arousal ratings being higher in the MDD than the HC group. Post-hoc investigation of these group effects showed that MDD subjects showed significantly higher arousal ratings before happy MIP (F(1,39) = 17.576, p<0.01) and a trend to higher ratings after sad MIP (F(1,39) = 6.735, p<0.056). None of the other ratings were significantly different between the groups (F’s (1,39)<3.0; p’s>0.05) (**Figure 3a,b**). These results suggest that when the brief temperatures were applied groups were not significantly different in their subjective arousal.

### Intensity and Affective Pain Thresholds: Evidence of “emotional allodynia”

**Figure 4:**
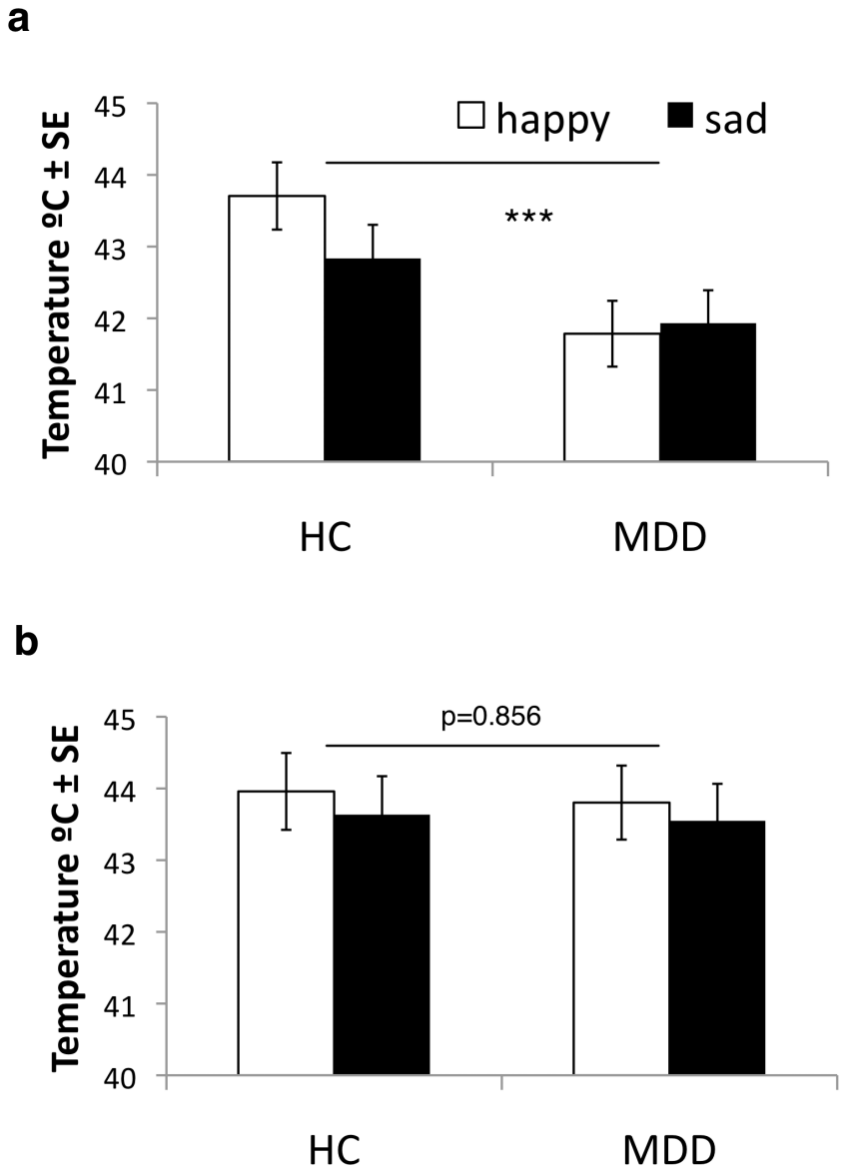
Pain Intensity and Affective Thresholds. **a)** Highly significant group effect on the affective thresholds was observed following happy and sad mood inductions whereby significantly lower affective thresholds were found in the MDD compared to the HC group (F(1,39) = 7.473, p<0.01); **b**) No significant group effect was observed for the intensity thresholds following mood induction (F(1,39) = 0.33, p>0.05).

Two-way repeated measures ANOVAs were performed on subjects’ intensity and affective pain thresholds to examine whether the groups differed in these measures following mood induction, a finding we have observed previously at baseline moods [19]. Highly significant group effect was found on the affective thresholds (F(1,39) = 7.473, p<0.01) whereby affective thresholds were significantly lower in the MDD compared to the HC group. No significant affective threshold by group interaction was observed(F(1,39) = 1.586, p>0.05)(**Figure 4a**). Unlike the affective thresholds, we found no significant group (F(1,39) = 0.33, p>0.05) or interaction effects (F(1,39) = 0.11, p>0.05) for the intensity thresholds (**Figure 4b**). Finally, the two-way repeated measures ANOVAs were performed on subjects’ intensity and affective thresholds in each group separately to examine whether sensory and affective thresholds differed, a finding we have observed previously in the MDD group, whereby affective pain thresholds were significantly lower than the pain intensity thresholds [19]f. Highly significant effect of rating was observed once again for the MDD group consistent with our prior findings(F(1,20) = 8.052, p<0.01), i.e., unpleasantness thresholds were significantly lower than the pain intensity thresholds in the MDD group. This was not significant for the HC group whereby intensity and unpleasantness thresholds were comparable (F(1,20) = 2.654, p>0.05).

## Discussion

We found that MDD subjects compared to healthy matched controls had lower pain unpleasantness thresholds, but similar pain intensity thresholds, to brief temperature stimuli across mood induction conditions. We also found that within the MDD group, but not the HC group, the unpleasantness thresholds were significantly lower than the corresponding pain intensity thresholds. Specifically, non-painful temperatures were consistently unpleasant for individuals with MDD irrespective of the induced mood. Taken together these results support the hypothesis that chronic mood state largely dictates affective pain experience, and raises the question as to whether emotional allodynia is a robust and pervasive characteristic of depression.

We found significantly decreased pain unpleasantness (affective) thresholds in the MDD compared to HC group following acute sad and happy mood induction. This finding is consistent and extends our prior study where we found that unmedicated MDD subjects showed decreased affective and sensory pain thresholds to brief temperature stimuli compared to age-, gender- and education-matched non-depressed comparison subjects [19]. Though there exist conflicting reports [6], [7], [9]–[11], [33], multiple authors describe increased sensitivity to experimental pain in MDD, especially when the affective-motivational or emotional dimension of pain is evaluated separately from the sensory-discriminative dimension [12]–[19]. This abnormal response in the affective dimension of pain was further supported by our observations of significantly decreased unpleasantness thresholds compared to the pain intensity thresholds in the depressed group, which is again consistent with our prior study [19]. The current findings suggest once again that the depression is associated with increased emotional responsivity to sensory stimulation, which need not be perceived as painful [19]. Importantly, by manipulating the acute mood in our participants through mood induction, we have shown for the first time that this increased affective responsivity to sensory stimulation, or emotional allodynia, in MDD is not just a reflection of the acute mood state and lower baseline affect in this disorder [34], [35]. Rather, emotional allodynia appears to probe the more stable aspects of depression that are independent of the acute mood state.

We may look to findings in neurophysiology to identify possible underpinnings of emotional allodynia in depression. Pain transmission by way of small diameter unmyelinated fibers (C-fibers) may be the pathway responsible for the stability of disordered pain response in depression. Several lines of evidence now point to the observation that afferent activation of C-fibers is particularly enhanced when one is depressed. For example, decreased cold-pressor tolerance [13], [14], [36], increased sensitivity to ischemic pain [15], [16], increased thermal postischemic paresthesias [37], and increased temporal summation of pain [17] have been repeatedly described in depression. All of these sensations are characterized by a strong affective component of pain that is conveyed via C-fibers. The small-diameter sensory C-fibers can be viewed as homeostatic, based on the idea that these afferents constantly relay information about the tissue status (reviewed in [38]). The fact that acute mood manipulations did not influence emotional allodynia in our depressed subjects is consistent with a homeostatic dysfunction in MDD [39] characterized by abnormal cortical interpretation of the homeostatic afferent C-fiber activity. Further reinforcing this notion are findings from a recent study that found increased perception of thermal grill illusion in depression [18]. Thermal grill illusion is an unpleasant burning sensation elicited by simultaneously applying adjacent non-noxious warm and cool stimuli on the skin [40]. It is related to C-fiber polymodal HPC (heat, pinch, cold) spinothalamic channel activity [41]. Just like emotional allodynia, the thermal grill is a central interpretation of the non-noxious peripheral activation of the thermosensory system [42], and it is enhanced in those who are depressed. Therefore, it is plausible that such abnormal cortical integration and interpretation of homeostatic messages in depression may underlie increased vulnerability to increased pain symptoms in MDD patients [43], [44]. This may also suggest that emotional allodynia may interfere with other homeostatic processes when one is depressed. Curiously, eating and sleeping behaviors change dramatically during a major depressive episode; perhaps, by interfering with these homeostatic processes, depressed subjects try to minimize the impact of their emotional allodynia. It will be important to examine whether depressed individuals that show more somatic symptoms also demonstrate more emotional allodynia.

We found that heat pain intensity thresholds were not statistically different between the MDD and HC groups following both sad and happy mood induction. This differs from findings in group differences in baseline moods, where sensory pain thresholds were also lower in MDD individuals [19], but is consistent with other mood induction studies [22]. Therefore, by succeeding in making non-depressed control subjects acutely more depressed with the sad MIP, and making MDD subjects acutely less depressed with the happy MIP, the sensory aspects of brief temperature stimuli were perceived in the same way irrespective of whether or not the subject was suffering from current MDD. Furthermore, these results were not confounded by differences in the arousal levels or medication [22], or by differences in mood induction susceptibility. Therefore, these findings suggest that baseline mood influences sensory appraisal in the depressed participants. Thus, future studies examining pain sensitivity in psychiatric disorders should use caution when employing sensory pain thresholds as their main variable to identify group differences.

In conclusion, this is the first report demonstrating the effects of acute sad and happy mood inductions on sensory and affective pain thresholds in unmedicated MDD individuals. Our results show that “emotional allodynia”, i.e., a qualitatively altered negative emotional response to normally non-aversive thermal stimuli, may be a chronic characteristic of those with MDD. By using both happy and sad mood induction in a group of unmedicated MDD subjects, as well as the healthy controls, we believe we have contributed evidence of decreased affective pain thresholds being a situationally robust feature of MDD. Therefore, our findings do not only further reinforce abnormal affect processing in MDD, but also point to this abnormality as a strong behavioral marker of those currently depressed. Further, this marker is not easily manipulated by affective state changes. Perhaps treatments aimed at minimizing emotional allodynia may prove to be more effective for those suffering from depression. Curiously, ketamine preferentially reduces pain affect [45], thermal grill illusion [46], and has shown remarkable effects in treatment-resistant depression [47]. Future studies should investigate whether individuals recovered from depression demonstrate emotional allodynia, or how pharmacological and/or psychological treatments influence this measure. Potentially interesting will be to examine this in treatment resistant depression and determine whether emotional allodynia is a notable characteristic of these individuals that contributes to the intractable nature of their symptoms. Similarly, future studies may wish to determine the degree to which emotional allodynia predicts the development of chronic pain complaints in those with MDD.

## Supporting Information

File S1 Details on mood induction procedure and music selection.
(DOCX)
