# Supplementary material for: Further Evidence of Emotional Allodynia in Unmedicated Young Adults with Major Depressive Disorder
Source: PLoS One. 2013 Nov 28;8(11):e80507. doi: 10.1371/journal.pone.0080507 (PMC3842925; doi:10.1371/journal.pone.0080507)
Supplement: File S1 — Details on mood induction procedure and music selection. (DOCX) [file pone.0080507.s001.docx]

*Induction Procedure*

Prior to psychophysical testing subjects wrote four autobiographical narratives and rated musical pieces. Each subject was asked to describe two very happy and two very sad personal events, each about a paragraph in length. Subjects rated each of these events on a scale from +1 (neutral) to +9 (extremely happy/sad) and were encouraged to select only events that had a happy/sad rating of 5 or higher. All subjects were also asked to listen to brief (30 sec) clips of 6 sad, 1 neutral and 6 happy musical selections *(*See **Table 1S**). Subjects rated each musical clip on a computerized VAS from -10/“Extremely Sad” to +10/“Extremely Happy” with 0/“Neutral” as the midpoint. The two highest rated happy/sad music pieces were then combined with happy/sad autobiographical scripts, respectively, to produce four total individualized MIPs.

After each mood induction, subjects were asked to rate their mood and arousal from: a) -10/“Extremely Sad” to +10/“Extremely Happy” with 0/“Neutral” as the midpoint; and b) -10/“Extremely Calm” to +10/“Extremely Aroused” with 0/“Neutral” as the midpoint, respectively. In addition, subjects used paper VAS before and after each mood induction block in order to measure their baseline mood before each induction, and to ensure that the mood returned to baseline level before second induction (**Figure 1**). The following paper VAS were used: a) -10/“Extremely Sad” to +10/“Extremely Happy” with 0/“Neutral” as the midpoint; and b) -10/“Extremely Calm” to +10/“Extremely Aroused” with 0/“Neutral” as the midpoint.

| **Table 1S: Music Selections** | | | | |
| --- | --- | --- | --- | --- |
| Title | | Composer/Artist | Album | |
| **Sad** | | | | |
| Adagio in G minor | Tomaso Albinoni | | |  |
| Drive Home | James Horner | | | Field of Dreams Soundtrack |
| Russia Under the Mongolian Yoke (played at half speed) | Sergei Prokofiev | | | Alexander Nevsky Soundtrack |
| Adagio for Strings, Op. 11 | Samuel Barber | | | Platoon Soundtrack |
| String Quartet No. 14 Op. 131, Adagio ma non tropo | Ludvig van Beethoven | | |  |
| Peer Gynt Suite Nos. 1-2, The Death of Ase | Edward Grieg | | |  |
| **Neutral** | | | | |
| Pocket Calculator | Kraftwerk | | | Computer World |
| **Happy** | | | | |
| Baroque & Blue, Suite for Flute and Jazz Piano | Claude Bolling & Jean-Pierre Rampal | | |  |
| This Old Train | Scott Cushnie, with Doug Riley and Joan Besen | | | Two Pianos, No Waiting |
| Nuns for Nixon | Bela Fleck with the New Grass Revival | | | Deviation |
| Asturian Way | Flook | | | Haven |
| Plekete | Zap Mama | | | Adventures in Afropea 1 |
| Signe | Eric Clapton | | | Unplugged |
|  |  | | |  |
